# Detecting and monitoring dental plaque levels with digital 2D and 3D imaging techniques

**DOI:** 10.1371/journal.pone.0263722

**Published:** 2022-02-15

**Authors:** Katja Giese-Kraft, Katja Jung, Nadine Schlueter, Kirstin Vach, Carolina Ganss

**Affiliations:** 1 Department of Conservative and Preventive Dentistry, Dental Clinic of the Justus-Liebig-University Giessen, Giessen, Germany; 2 Division for Cariology, Department of Operative Dentistry and Periodontology, Center for Dental Medicine, Medical Center, University of Freiburg, Faculty of Medicine, University of Freiburg, Freiburg, Germany; 3 Institute of Medical Biometry and Statistics Faculty of Medicine and Medical Center - University of Freiburg, Freiburg, Germany; Centre Hospitalier Regional Universitaire de Tours, FRANCE

## Abstract

Detecting and monitoring dental plaque is an important issue in research and clinical practice. In this context, new digital imaging methods that permit permanent documentation of the clinical findings could be promising tools. The aim of the study was therefore to investigate whether disclosed plaque can be reliably visualised on 2D and 3D images captured with digital intraoral imaging devices. Clinical examination was the reference method. Twenty subjects (27.5±1.2 years) were included and plaque was measured at three different stages: habitual plaque (T1), after 72 h without oral hygiene (T2) and after a subsequent habitual brushing exercise (T3). At each time point, plaque was disclosed followed by the clinical examination and capturing the 2D and 3D images (intraoral-camera CS 1500 and intraoral-scanner CS 3600; Carestream Dental, Germany). Plaque amounts were recorded on oral and vestibular surfaces of the Ramfjord-teeth (16, 21, 24, 36, 41, 44) using the Rustogi-modified-Navy-Plaque-Index (RMNPI) and expressed as percentage of plaque-containing RMNPI areas of all RMNPI areas. At T1, percentages (mean±SD) obtained from the clinical examination, 2D and 3D images were 62.2±10.6, 65.1±10.0 and 64.4±10.6 resp. increasing to 76.9±8.0, 77.9±8.6 and 77.5±9.4 resp. at T2. After toothbrushing (T3), values decreased to 56.3±11.1, 58.2±12.1 and 61.2±10.8 resp. All methods were able to show statistically significant changes in plaque amounts at the different time points with in part statistically significant but minor differences between them. The Bland-Altmann analysis revealed a good agreement between values from both 2D and 3D images with the clinical examination. The agreement of the scores obtained with the both image-based methods for the single RMNPI areas with the clinical examination was mainly classified as substantial to almost perfect. Amounts of plaque can be reliably detected and monitored on 2D images from an intraoral camera and on 3D images from an intraoral scanner.

## Introduction

Dental caries and periodontal diseases are plaque-associated conditions with high global prevalence [1]. Therefore, among other measures, achieving adequate oral hygiene levels is an urgent objective in prevention programmes as well as in individual oral health education. Part of such efforts is detecting and quantifying plaque and monitoring plaque levels. The most commonly used procedure is measuring plaque levels using clinical index systems [2, 3]. Mostly, plaque is disclosed and the amount of which is clinically recorded and scored in defined areas of the tooth. This approach has a long tradition, but a major disadvantage is that the clinical examination is not image-based. This means that the result can only be documented by the scores that are assigned chairside. Recoding into other indices, e.g. in order to make results comparable to other data sets, or addressing new research questions at a later point in time is therefore only possible to a very limited extent. Furthermore, the result depends on the current examination quality and cannot be repeated. It would therefore be desirable to have an image-based procedure that allows a permanent documentation of an oral hygiene status.

In the context of plaque detection, intraoral images were mostly taken with extra-oral cameras. Respective applications are the detection of plaque on fluorescence images (e.g. [4, 5]) or the planimetric evaluation of conventional images [3]. However, taking images of the entire dentition is very time-consuming and technically demanding. Furthermore, such images are often not evenly illuminated and the proximal areas in particular are not adequately visualised due to the curvature of the teeth. Intraoral cameras may be much better suited for this purpose, as they can be easily positioned at a favourable angle to the tooth axis and illuminate the object without flash.

A relatively novel approach is 3D imaging with intraoral scanners. Such devices have many applications in dentistry [6] and respective 3D data have been also used for diagnostic purposes e.g. monitoring tooth wear [7, 8]. Scanners are able to create colour 3D images in relatively short time and might be therefore a promising tool for detecting and monitoring dental plaque levels. Besides saving time, a major advantage of 3D imaging is that all areas of the tooth can be visualised very easily by rotating the object. Earlier studies already investigated differences between a digital camera versus an intraoral camera in terms of plaque imaging reliability, but there is just little known about this topic yet and further investigation have to be done [9]. So far, however, there is little work done on plaque measurements with 2D or 3D imaging techniques, but there is first evidence [10, 11] that these techniques are very promising.

The aim of the present study was therefore to investigate, whether plaque can be reliably detected, quantified and monitored on images obtained by the both intraoral imaging techniques. The method of comparison was the clinical examination.

The Rustogi modification of the Navy-Plaque Index (RMNPI) [12] was used and the plaque levels investigated were i) habitual, ii) after abstaining from oral hygiene for 72 h and iii) after a habitual toothbrushing procedure after this 72 h period.

## Subjects, materials and methods

The present study is a prospective in vivo study and was approved by the Ethics Committee of the Justus-Liebig-University, Giessen, Germany (Doc. No 142/19). The consent was obtained in written form.

It took place at the Department of Conservative and Preventive Dentistry, Dental Clinic of the Justus-Liebig-University Giessen, Germany and was performed according to the guidelines of Good Clinical Practice and the Declaration of Helsinki.

The study group was a convenience sample and consisted of 20 subjects (16 female, 4 male) with a mean (±SD) age of 27.5±1.2 years, recruited via oral and written announcements. Inclusion criteria were: written informed consent, at least 18 years of age, full dentition up to the second molar without extensive fillings on vestibular and / or oral surfaces or prosthetic restorations, good general health based on their medical history, agreement to return for their scheduled visit and to follow all study procedures. Exclusion criteria were: frank carious lesions, insufficient fillings, dental malformations, gingival recessions >1/3 of the root length in the region of interest, active treatment for periodontitis, fixed orthodontic appliances (retainer permitted), or any disease or condition that could be expected to interfere with examination procedures or with the subject safely completing the study.

### Study procedures and 2D / 3D imaging process

In order to ensure that different levels of plaque amounts could be detected, data were collected at the first appointment when subjects came with habitual plaque levels (T1; baseline), at the second appointment after 72 hours without oral hygiene (T2) and again after subsequent habitual toothbrushing (T3). All procedures were highly standardized and have been performed in the same order for all subjects.

At the first visit, informed consent was obtained and subjects were screened for inclusion / exclusion criteria. After inclusion in the study (T1), dental plaque was disclosed with Mira-2-Ton^®^ solution (Hager & Werken GmbH & Co KG, Duisburg, Germany). The disclosing agent was applied with a saturated foam pellet, thereafter the subjects rinsed with water for 10 s. This disclosing procedure was done twice. Immediately afterwards, plaque levels were recorded on the vestibular and oral surfaces of the Ramfjord-teeth (16, 21, 24, 36, 41, 44) [13] using the Rustogi modification of the Navy-Plaque Index (RMNPI). After completing the clinical examination, intraoral 2D images and intraoral 3D scans (intraoral camera CS 1500 and intraoral scanner CS 3600 Carestream Dental LLC, Atlanta, United States and Stuttgart, Germany) were done. 2D images were taken using a dental air blower and the application of a lip retractor (OptraGate; Ivoclar Vivadent, Schaan, Liechtenstein) for full display. Four intraoral 2D images were taken from each of the teeth under study: from a mesial-lateral and distal-lateral view of both the vestibular and oral surface.

**Fig 1:**
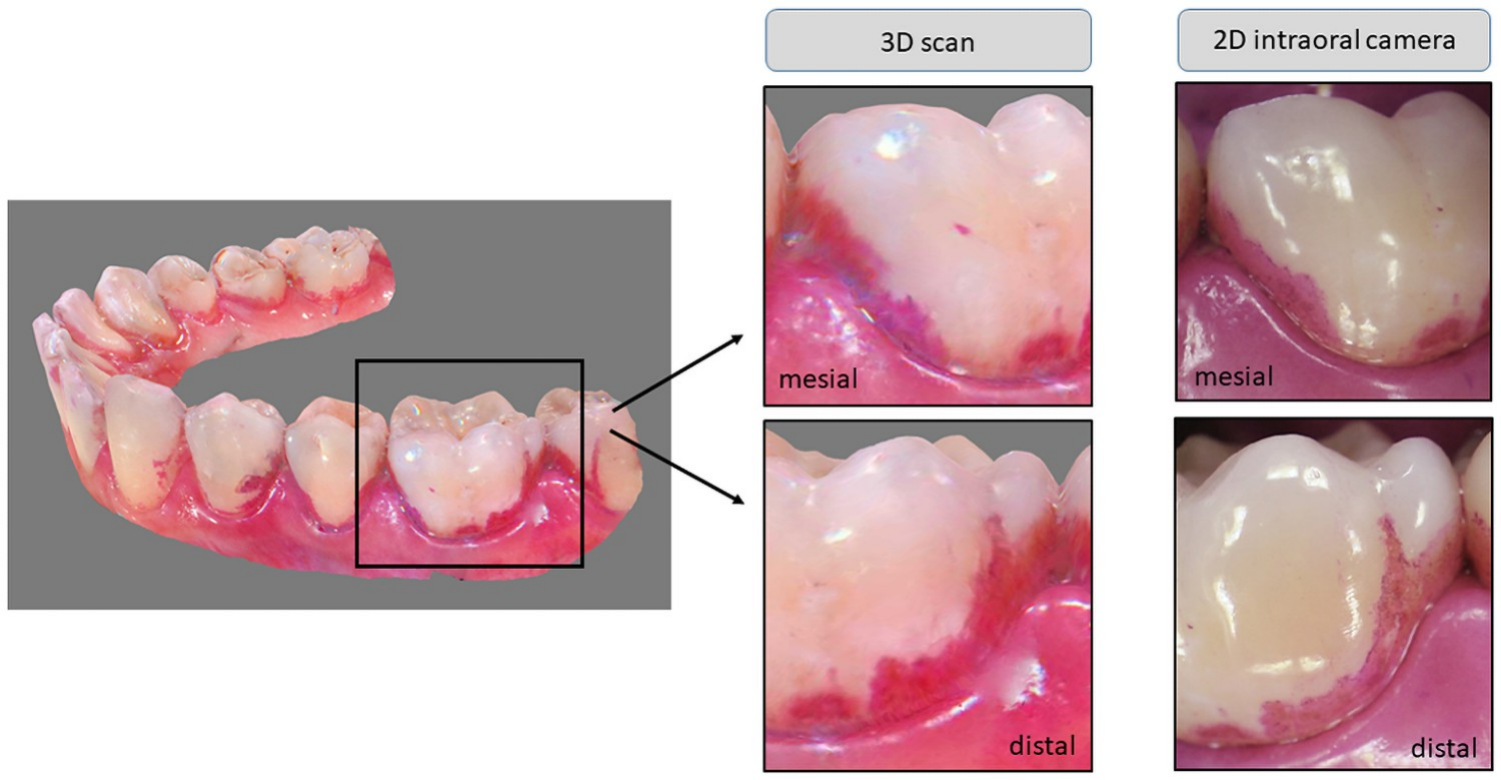
Example of a 3D scan and the associated 2D intraoral images. The left side shows a completely scanned lower dental arch, the right side excerpts of this 3D scan in a mesial-lateral and distal-lateral view of tooth 36 with the associated 2D intraoral images at baseline (T1).

Then an intraoral 3D-scan of the whole dentition was made, again with the lip retractor in situ. The scan area was kept dry by sucking off saliva and using an air blower. Areas that were not sufficiently well visualised were re-scanned. On the one hand, these were areas of the dental arch, not captured immediately during the scanning process (shown as “black holes” in the scan-image). On the other hand, it occurred that some areas, even if they appeared to be completely scanned at first, were shown as obviously not captured “blue areas” after the computing process. This was especially the case in proximal areas. Therefore, rescanning was done until the entire dental arch was completely displayed. Fig 1 shows an example of a completely scanned dental arch and the associated mesial-lateral and distal-lateral 2D intraoral images.

**Fig 2:**
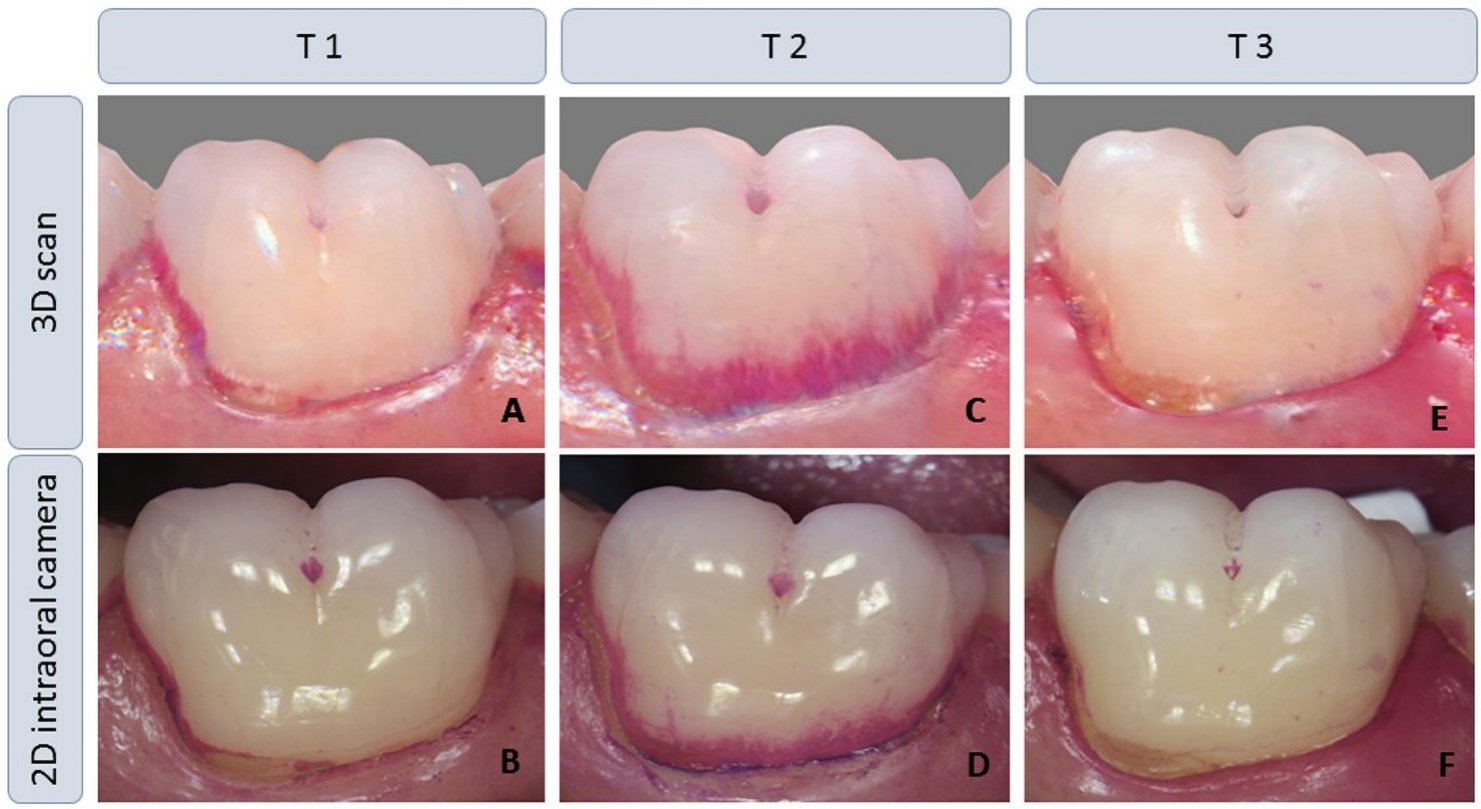
Different degrees of plaque amount recorded with 3D scan and 2D intraoral camera at the three time points (T1, T2, T3). A, C, E: 3D scan of the vestibular surface of a tooth 36 and B, D, E the associated 2D intraoral image; A, B: habitual amount of plaque (T1); C, D: amount of plaque after 72h without oral hygiene (T2); E, F: amount of plaque after brushing (T3).

Finally, the teeth were cleaned with pumice and a rubber cup until they were free from disclosed plaque. After 72 hours without oral hygiene (T2), plaque was again disclosed and the clinical examination and 2D and 3D imaging were done, all as described above. Next, subjects brushed their teeth depending on their preferred toothbrush type either with a manual toothbrush (elmex^®^ sensitive, GABA GmbH, Swiss, Colgate Palmolive Company, USA) or with a powered toothbrush (Professional Care 3000 oral-B^®^, powered by BRAUN GmbH, Germany, Procter & Gamble, USA) using toothpaste (blend-a-med^®^ PRO EXPERT, blend-a-med-Forschung, Germany, Procter & Gamble, USA) and as they habitually do. Afterwards, the disclosing and imaging procedures were done a third time (T3). Finally, a second professional tooth cleaning completed the clinical study procedures. Fig 2 shows illustratively different degrees of plaque amount recorded with 3D scan and 2D intraoral camera at the three time points (T1, T2, T3) on the vestibular surface of a tooth 36.

### Plaque-index

The RMNPI was determined for each Ramfjord-tooth during the clinical examination and afterwards on both 3D and 2D images. The vestibular and oral tooth surfaces of the Ramfjord-teeth were investigated and each of the nine RMNPI areas were scored 0 (no disclosed plaque visible) or 1 (any amount of disclosed plaque visible). The plaque index value was calculated as percentage of areas with plaque of the total areas.

**Fig 3:**
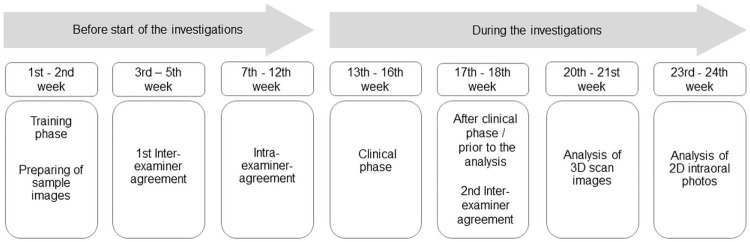
Timeline of the study procedure. Overview of the chronological sequence of the evaluations, including the determination of inter- and intra-examiner agreement.

The judgement of the 3D and 2D images was carried out after the clinical examination was totally completed, with the 3D and 2D images being assessed separately from one another. An overview of the chronological sequence of the evaluations, including the determination of inter- and intra-examiner agreement, is shown in the timeline-chart below (Fig 3). Because of time lags and the large number of decisions, that had to be made, it was impossible for the two investigators to relate 3D and 2D images to one another; a randomization of the images for analysis was not performed.

All images were evaluated using CS Mesh Viewer (Carestream Dental, Germany) and the same 15.6 inch monitor (PnP monitor, Intel HD Graphics 630, model N850EP6, MIFCOM GmbH, Germany). The graphic display showed a 1920 x 1080 resolution (Full-HD), an 8-bit colour depth, an RGB Standard Dynamic Range colour space and a 120 Hz screen refresh rate. The display brightness was set to 50% and the scaling to the recommended 125%.

### Inter- and intra-examiner agreement

All procedures and conversations with the subjects were standardized. The study was carried out by two investigators (K.G-K, K.J), who were extensively trained and calibrated before and during the investigation. In order to maintain a consistent evaluation, sample images of one anterior, one premolar and one molar each were taken with the RMNPI areas marked on them, and these images were available for each evaluation. The inter- and intra-examiner agreement was determined prior to the clinical phase of the study (on three subjects not involved in the study); after the clinical phase, prior to the analysis of the images, the inter-examiner reliability was determined a second time (on two randomly chosen subjects participating in the study) (Fig 3). Kappa scores were calculated for the single areas of the RMNPI of each of the six teeth under study and are presented here as mean±SD (Table 1). This was done separately for all study time points (T1-T3).

**Table 1:** Inter- and intra-examiner reliability as mean±SD of the Kappa scores of the different areas of the index per time points T1-T3.

	T1	T2	T3	T1	T2	T3
	Calibration 1			Calibration 2		
Inter-examiner						
CE	0.853±0.090	0.957±0.065	0.914±0.105			
2D	0.852±0.226	0.762±0.200	0.912±0.064	0.830±0.110	0.890±0.101	0.742±0.165
3D	0.850±0.176	0.745±0.235	0.720±0.128	0.834±0.134	0.854±0.133	0.767±0.192
Intra-examiner						
Examiner 1						
2D	0.815±0.234	0.793±0.274	0.878±0.119			
3D	0.781±0.185	0.797±0.204	0.819±0.121			
Examiner 2						
2D	0.782±0.310	0.900±0.138	0.948±0.070			
3D	0.960±0.041	0.880±0.150	0.966±0.054			

CE: clinical examination, 2D: images from the intraoral camera, 3D images from the intraoral scanner. Calibration 1: prior to the clinical study phase, calibration 2: after finalising the clinical phase and prior to the analysis of the images.

### Statistics

Statistics were done with Stata (StataCorp LT, College Station, TX, USA, Version 16.1) and with IBM SPSS Statistics version 25 (IBM Germany GmbH, Ehningen, Germany). As there were no significant deviations from the Gaussian distribution (Kolmogorov-Smirnov-test), parametric test procedures were used for all statistical analyses. Values are given as mean ± standard deviation (SD).

For each participant, the amount of plaque was expressed as the percentage of the RMNPI areas containing plaque of all areas. Values are given as mean ± standard deviation (SD). To investigate, whether all methods were able to detect differences in the plaque amounts at baseline, after abstaining from oral hygiene and after toothbrushing (within-methods comparisons), a linear mixed model was used. To investigate, whether there is a difference between amounts of plaque detected at each of the three time points by the different methods (between-method comparisons) a linear mixed model was applied, too. The method of Scheffe was used to correct for multiple testing in case of pairwise comparisons.

Percentages for the oral and vestibular surfaces of each tooth are presented only descriptively.

Bland-Altman-plots [Bland and Altman, 1999; Giavarina, 2015] were used for analysing the agreement of plaque amount values obtained from 2D and 3D images in comparison to the clinical investigation as the reference. This was done for oral and vestibular surfaces separately. A regression analysis was performed to evaluate whether the order of values has an effect on agreement (proportional bias). One-sample t-tests of the mean differences of values obtained from the two methods with values from the clinical investigation were made to evaluate whether there is a systematic bias.

Further, the 9 areas of the RMNPI were regarded separately and the agreement of the results from the different methods was calculated with Kappa statistics.

## Results

### RMNPI scores

Table 2 shows the percentages of plaque containing areas obtained from the different methods at baseline, after abstaining from oral hygiene and after habitual toothbrushing.

**Table 2:** Percentages of plaque containing RMNPI areas of all RMNPI areas (mean (SD)).

	T1			T2			T3		
	CE	2D	3D	CE	2D	3D	CE	2D	3D
**All surfaces**	62.1 (10.6)	65.1^a^ (10.0)	64.4^Ba^ (10.6)	76.9^a^ (8.0)	77.9^a^ (8.6)	77.5^a^ (9.4)	56.3^a^ (11.1)	58.2^a^ (12.1)	61.2^B^ (10.8)
**Vestibular surfaces**	60.2 (14.5)	64.0^a^ (13.6)	62.9^Ba^ (13.6)	81.2^a^ (9.0)	83.0^b^ (9.5)	82.0^ab^ (9.4)	53.0 (13.3)	56.2^a^ (15.0)	58.5^Ba^ (13.6)
**Oral surfaces**	64.1^Ac^ (11.8)	66.2^a^11.7	65.8^Bac^ (11.6)	72.5^a^ (9.3)	72.9^a^ (9.9)	73.0^a^ (11.8)	59.7^Aa^ (11.9)	60.3^a^ (11.7)	63.8^B^ (10.8)

T1: baseline, T2: after abstaining from oral hygiene for 72 h, T3: after habitual toothbrushing (T3). CE: clinical examination, 2D: image obtained from the intraoral camera; 3D: images obtained from the intraoral scanner. The same upper case letters indicate no significant difference between time points (within-methods methods); the same lower case letters indicate no significant differences within time points (between-methods comparison). Level of significance <0.05.

The clinical investigation revealed a significant increase of plaque amounts when subjects abstained from oral hygiene compared to baseline (p<0.0001). After subjects had brushed their teeth, amounts decreased significantly (p<0.0001) to somewhat lower plaque levels than baseline (p<0.01). Similar was detected on both 2D and 3D images: plaque amounts increased without oral hygiene (each p<0.0001 compared to baseline), and decreased significantly after brushing (each p<0.0001 compared to T2). Post brushing plaque levels on 2D images were slightly lower compared to baseline (p = 0.001) while there was no significant difference of plaque amounts between these time points on 3D images (n.s; p = 0.125).

With respect to the different surfaces, the changes in plaque amount at the different time points were more pronounced on the vestibular surfaces than on the oral surfaces. These differences were found both on clinical examination and after analysis of the 2D and 3D images (Table 2).

At the different time points, there were few differences between values obtained from the different methods. These were of minor order though they reached in part statistical significance (Table 2).

**Fig 4:**
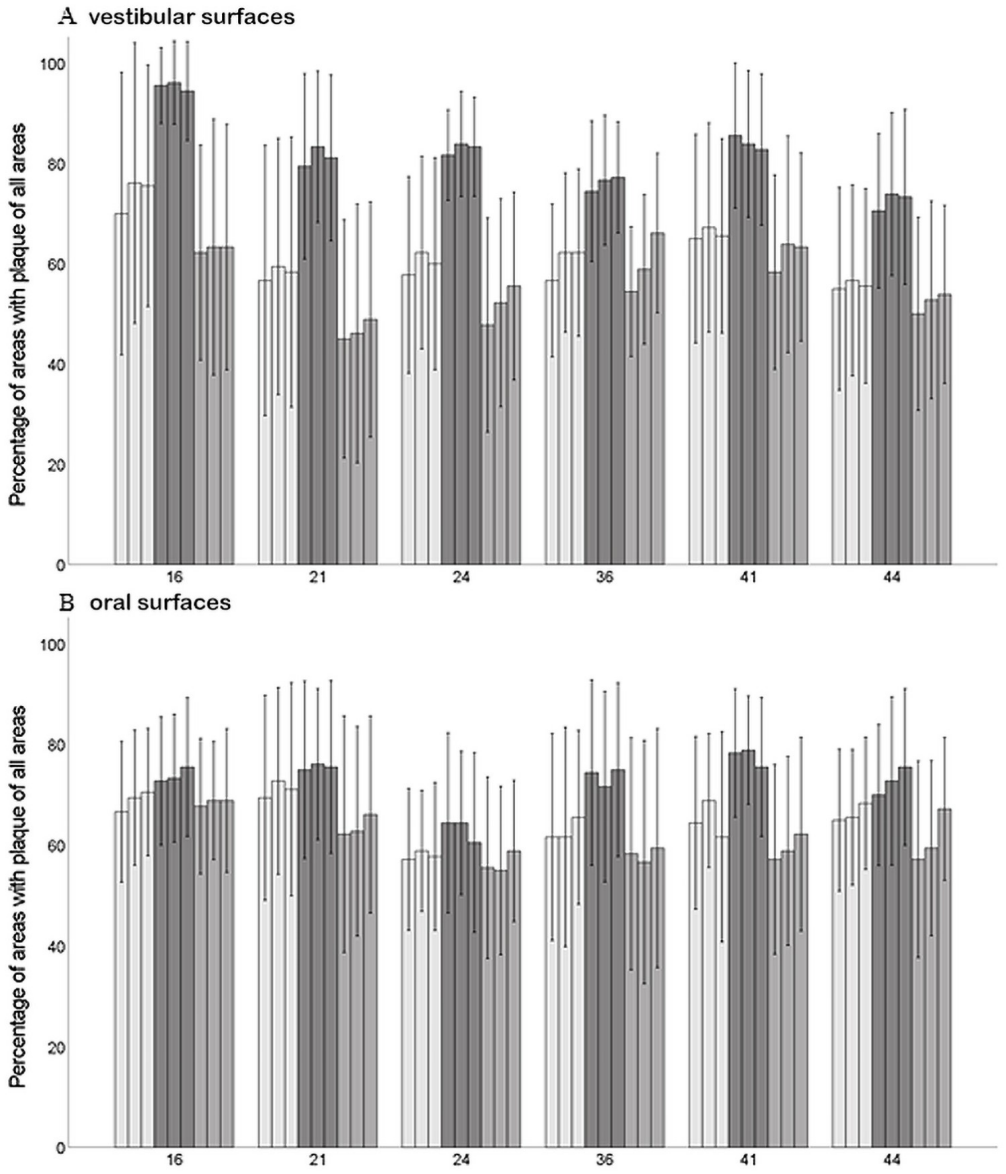
Percentages of plaque containing RMNPI areas (mean±SD). 16 and 36: upper right and lower left first molar, 24 and 44: upper left and lower right first premolar, 21 and 41: upper left and lower right first incisor. Light grey columns: T1 (habitual oral hygiene level); dark grey columns: T2 (after abstaining from oral hygiene for 72 h); medium light grey: T3 (after habitual brushing). Within a time point: left column: clinical investigation; middle column: 2D images; right column: 3D images.

The amounts of plaque for the vestibular and oral surfaces of the individual teeth is shown in Fig 4. The numerical increase of scores after abstaining from oral hygiene was most pronounced on vestibular surfaces in particular on the first upper molar (Fig 4A), while changes in plaque scores on the oral surfaces at the different time points are relatively small (Fig 4B).

**Fig 5:**
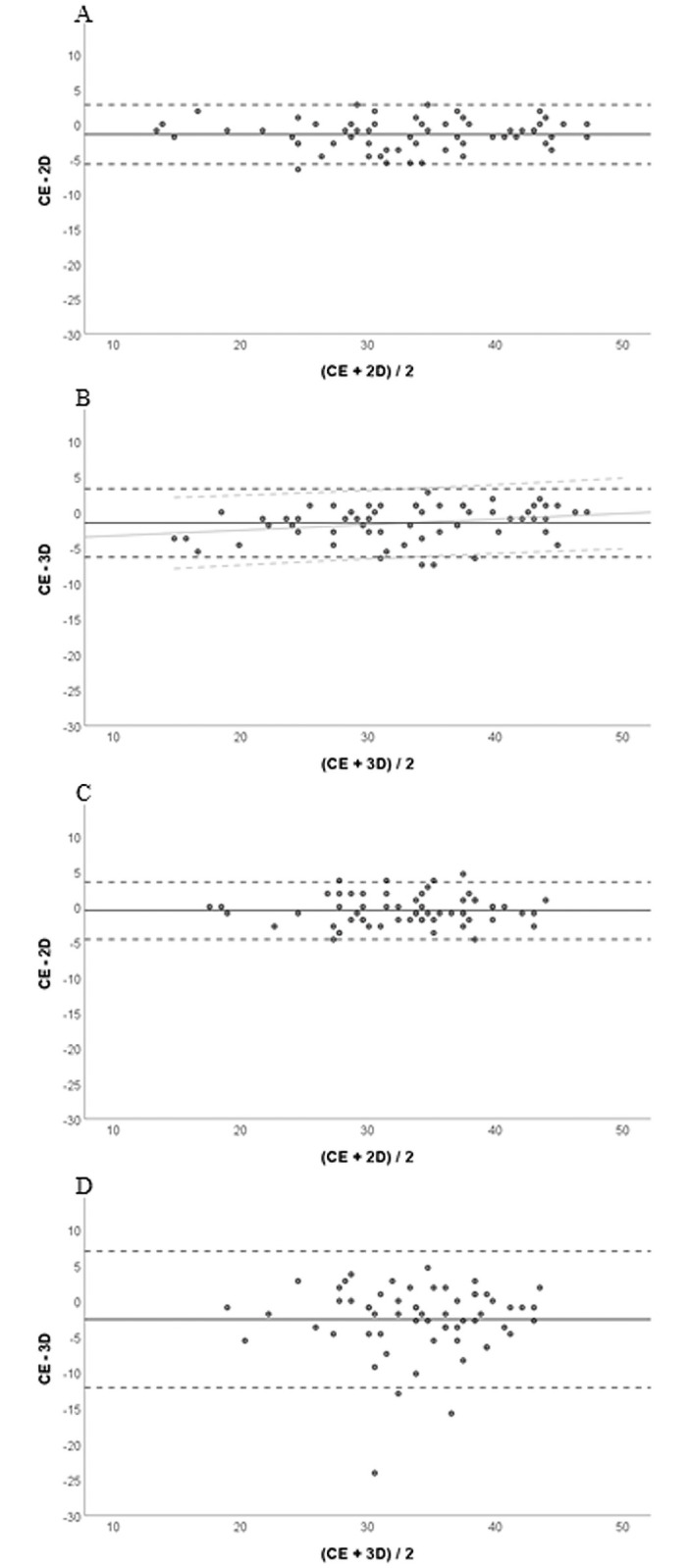
Bland-Altman plots evaluating the agreement between the percentages of plaque containing RMNPI areas obtained from 2D and 3D images with the clinical evaluation. A and B: vestibular surfaces, C and D: oral surfaces. The solid line indicates the mean difference of the methods of comparison; the broken lines indicate the 95% limits of agreement (mean±1.96xSD). The light grey lines in B show the regression line with 95% confidence intervals indicating a significant albeit small proportional difference, which was not found in A, C and D.

The Bland-Altmann analyses (Fig 5) revealed a small systematic bias as slightly higher scores were found on 2D (except oral surfaces) and 3D images compared to the clinical examination. For oral surfaces, the mean difference to the clinical examination was -0.5±2.1 for 2D (n.s.; p = 0.064) and -2.6±4.9 for 3D (p<0.001) images. For vestibular surfaces respective values were -1.5±2.2 (p<0.001) and -1.5±2.4 (p<0.001). There was no proportional bias between the clinical examination and the two other methods except for 3D images of vestibular surfaces. Here, there was a minor but statistically significant tendency to higher scores at low plaque levels and lower values at higher plaque levels (r^2^ = 0.069; p<0.05).

### Agreement of methods within the RMNPI areas

Overall, according to Landis and Koch [14], the agreement between methods was substantial to almost perfect. The results of the evaluation of the 2D images agreed slightly better with the clinical examination than those of the 3D images. The results from the two image-based methods showed very good agreement (Table 3).

**Table 3:** Kappa coefficients with 95%CI in brackets for the individual areas of the RMNPI, all time points and areas are merged.

Area	Overall	CE-2D	CE-3D	2D-3D
A	0.648 [0.58, 0.70]	0.749 [0.68, 0.82]	0.555 [0.47, 0.64]	0.634 [0.54, 0.72]
B	0.811 [0.77, 0.84]	0.840 [0.80, 0.88]	0.764 [0.71, 0.82]	0.831 [0.78, 0.88]
C	0.664 [0.57, 0.74]	0.732 [0.65, 0.82]	0.614 [0.52, 0.71]	0.648 [0.55, 0.75]
D	0.680 [0.62, 0.78]	0.789 [0.70, 0.88]	0.606 [0.49, 0.72]	0.649 [0.54, 0.76]
E	0.898 [0.86, 0.92]	0.909 [0.87, 0.94]	0.864 [0.82, 0.91]	0.919 [0.89, 0.95]
F	0.739 [0.64, 0.79]	0.824 [0.75, 0.90]	0.659 [0.60, 0.76]	0.736 [0.64, 0.83]
G	0.756 [0.72, 0.80]	0.785 [0.74, 0.83]	0.682 [0.63, 0.74]	0.802 [0.76, 0.85]
H	0.726 [0.68, 0.76]	0.760 [0.71, 0.81]	0.647 [0.59, 0.70]	0.771 [0.72, 0.82]
I	0.770 [0.71, 0.82]	0.778 [0.73, 0.83]	0.689 [0.63, 0.75]	0.839 [0.80, 0.88]

Overall: coherence measure for all 3 devices; CE-2D: comparison of results from the clinical examination with results from the intraoral camera images, CE-3D: comparison of results from the clinical examination with results from the intraoral scanner image, 2D-3D: comparison of the results from the images from the intraoral camera and the intraoral scanner.

**Fig 6:**
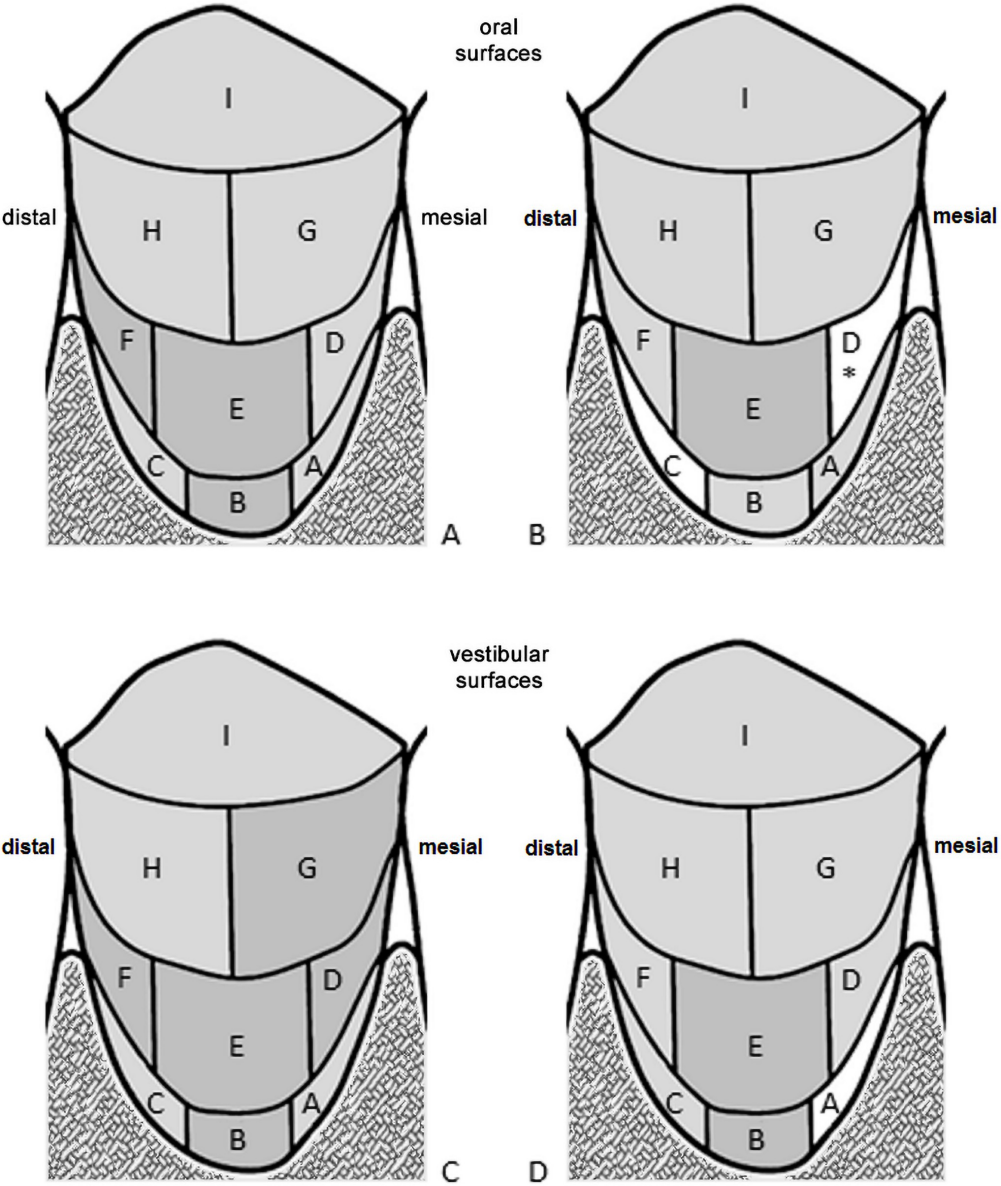
Agreement of the two image-based methods with the clinical examination in the areas of the RMNPI separated into the oral and vestibular areas. A and C: 2D images compared to the clinical examination, B and D: 3D images compared to the clinical examination; A and B: oral surfaces, C and D: vestibular surfaces. Areas A, D, G = mesial; areas C, F, H = distal. Strength of agreement according to [14]: dark gray: 0.81–1 (almost perfect), light gray: 0.61–0.8 (substantial), white: 0.41–0.60 (moderate). Asterisk: in this area, the kappa score was 0.269 (fair).

Similar results were found when the oral and vestibular regions of the dentition were regarded (Fig 6A–6D). Results from the 2D images showed for both oral and vestibular surfaces a substantial to almost perfect agreement to the clinical examination (Fig 6A and 6C) with the lowest Kappa score of 0.681 occurring on area D on oral surfaces. Results from the 3D images on vestibular surfaces (Fig 6D) agreed almost as well as those from the 2D analysis with the clinical examination. The lowest Kappa score here was 0.491 indicating a moderate agreement. On the oral surfaces (Fig 6B), the agreement of the 3D results with the clinical examination was substantial in the majority of cases, but only moderate in area C (κ = 0.506) and only fair in area D (κ = 0.269).

## Discussion

The study was designed to investigate, whether it is possible to detect and to monitor plaque levels on colour images obtained from an intraoral camera and an intraoral scanner in a reliable way. Plaque amounts were measured at habitual levels, after accumulation during a period of no oral hygiene and after a single habitual tooth brushing exercise. This was chosen to investigate, whether the method is not only able to detect plaque, but also whether it is able to monitor individual changes of plaque amounts with time. The RMNPI was used because it is one of the most complex indices investigating nine different areas of the tooth surface. The many areas of the RMNPI allow a very differentiated investigation of whether all areas of a tooth surface can be well imaged with the two imaging methods. In addition, the complex RMNPI can also be recoded into simpler indices, so that it also maps other index systems in a certain way. A disadvantage of the RMNPI is that it does not differentiate the amount of plaque within an area. Both areas with small amounts of plaque and areas that are completely covered with plaque receive the same code, making this index not very discriminative. We applied the RMNPI very strictly, which means that we only evaluated an area as negative if it was entirely free of stained plaque. In other words, as soon as even a small plaque island was present, the area was evaluated as positive. This would on one hand explain the relatively high RMNPI scores overall. On the other hand, the fact that we found comparably high RMNPI scores on the 3D as well as on the 2D images and in the clinical examination implies that small plaque islands can as well be diagnosed on intraoral scans. Otherwise, the scores on the 3D images should have been lower than on the other two examination methods.

A challenge with the RMNPI is that it is relatively difficult to use and requires extensive calibration. We therefore conducted several calibration sessions, and in addition, sample images were always available during the clinical examination and the evaluation of the images. Through these efforts, the strength of agreement was substantial to almost perfect for both the intra- and inter-rater agreement (Table 1).

During the preparation for the study, it was realised that a single 2D image could not capture the mesial and distal areas of a tooth equally well. Therefore, four images were taken of each tooth to image the proximal areas of the oral and vestibular surfaces in an optimal way. In order to limit the effort and time required by the participants, the investigation was done on selected teeth rather than on the whole dentition. Partial-mouth recordings have been widely used in particular in the field of periodontology [15]. One of the most often used approaches is using the Ramfjord teeth [13] which have been shown to be valid for representing plaque levels in age groups similar to those investigated here [16]. Ramfjord teeth cover both the right and left side of the dentition as well as both jaws and include all tooth types. Overall, our partial-mouth approach can be therefore regarded as sufficiently representative for the whole dentition and for our research question under study.

At baseline, the clinical investigation revealed that subjects presented with significant amounts of habitual plaque; 62.1% of the RMNPI areas contained plaque to a various extent. As expected, the amount of plaque increased after 72 hours without oral hygiene and reached a value of 76.9%. Corroborating findings from an earlier study [4], this increase was greater on the vestibular surfaces than on the oral surfaces and the numerically highest values were found on the upper first molar. Habitual brushing reduced the amount of plaque, but plaque coverage was only slightly lower than at baseline. Overall, the plaque scores were already relatively high at baseline and the changes at the different time points were not very pronounced. This may be mainly due to the fact that, as mentioned above, a positively evaluated area can be completely plaque-covered or have just a small plaque spot resulting in a limitation of discriminative power of the RMNPI.

The images obtained from the intraoral camera as well as from the intraoral scanner revealed a very similar result. As in the clinical examination, both methods showed a significant increase in the amount of plaque after abstaining from oral hygiene and a reduction after tooth brushing. Overall, there was a tendency to somewhat numerically higher scores on both 2D and 3D images compared to the clinical examination, which was also found in the Bland-Altman analysis. This can be explained by the fact that teeth on the images were enlarged compared to the clinical situation, the illumination of the areas under study was usually very good compared to the clinical situation and there were no patient-related factors, which would have made the evaluation difficult.

Compared to the 2D images, which had an excellent resolution and sharpness, the 3D images appeared somewhat pixelated and blurred, especially in proximal areas. It was also sometimes difficult to demarcate neighbouring teeth from each other. This led to a lower agreement of the plaque scores with the clinical examination in two gingival and one proximal area, but overall the agreement was still substantial.

Bland-Altmann analyses were performed to investigate how well the two image-based methods matched the clinical examination in individual cases. This revealed that percentages of plaque containing RMNPI areas found on 2D images agreed very well with the clinical results both for oral and vestibular surfaces. For the 3D images, the agreement to the clinical examination depended on the area examined. For the vestibular surfaces, the agreement was equivalent to that of the 2D images, but there was a statistically significant, albeit small, proportional error; results from 3D images may slightly overestimate the amount of plaque at low magnitudes and slightly underestimate it at large magnitudes. At least for the amounts of plaque observed here, the dimension of this bias does not seem relevant. However, it needs to be investigated in more detail whether intraoral scanners are also suitable for recording smaller amounts of plaque than those investigated here.

For the oral surfaces, there was no such proportional error, but the agreement with the clinical findings was clearly lower than for the vestibular surfaces. One reason for this is that due to the size of the scanner head, it can be difficult to display the oral surfaces of the mandible depending on the anatomical situation, so that the scanner software does not compile an ideal image. However, the software of the scanner used here offers the possibility to access the 2D images from which the 3D object is calculated. These images have a similar quality to those of the intraoral camera, so that another diagnostic option is available for areas that are not ideally represented on the 3D image. However, the intraoral scanners have developed rapidly so far and will certainly be able to provide even better images in the future, so that these problems can be solved.

A clear limitation of the study is that only younger people with few restorations were included. Plaque detection on 2D intraoral images should also be possible in all other clinical conditions, but 3D imaging with intraoral scanners may have limitations. For example, it is unclear how metallic restorations, such as extensive restorations with crowns, affect plaque detection. Furthermore, 3D imaging is limited in patients with recessions and wide interdental spaces, such as periodontal disease. Further studies must therefore investigate to what extent intraoral scanners are also suitable for plaque detection under these conditions.

Another limitation of our method evaluation is that we only used one index. Although the RMNPI is complex and can be recoded into other indices, the latter could nonetheless represent slightly different approaches. Therefore, further investigations with other index systems would certainly be very important.

## Conclusion

Amounts of plaque can be reliably detected and monitored on 2D images from an intraoral camera and on 3D images from an intraoral scanner when compared to the clinical examination as reference. Intraoral 2D images allow a more precise plaque determination, but taking and managing such images is relatively time-consuming. Intraoral 2D images are therefore more suitable for special research questions and for partial mouth recordings. Intraoral scanners can image the entire dental arch in a relatively short time and all areas can be easily assessed by rotating the 3D object. However, this is somewhat at the expense of image quality, at least so far. Nevertheless, intraoral scanners seem to be reliable enough to detect and monitor plaque in research and clinical practice, at least when it comes to the plaque levels studied here. They could therefore be promising tools for comprehensive analysis and for documenting the oral hygiene situation of patients in the future.

## Supporting information

S1 Data (XLSX)

## References

[1] Collaborators GBDOD, Bernabe E, Marcenes W, Hernandez CR, Bailey J, Abreu LG, et al. Global, Regional, and National Levels and Trends in Burden of Oral Conditions from 1990 to 2017: A Systematic Analysis for the Global Burden of Disease 2017 Study. J Dent Res. 2020;99(4):362–73. doi: 10.1177/0022034520908533 32122215 PMC7088322

[2] Fischman SL. Current status of indices of plaque. J Clin Periodontol. 1986;13(5):371–4, 9–80. doi: 10.1111/j.1600-051x.1986.tb01475.x 3013947

[3] Pretty IA, Edgar WM, Smith PW, Higham SM. Quantification of dental plaque in the research environment. J Dent. 2005;33(3):193–207. doi: 10.1016/j.jdent.2004.10.017 15725520

[4] Ganss C, Glanz A, Glanz T, Schlueter N, Ruf S. Red fluorescence of plaque in the dentition-a comparison of Quantitative Light-induced Fluorescence-Digital (QLF-D) images and conventional images of disclosed plaque. Photodiagnosis Photodyn Ther. 2020;32:102063. doi: 10.1016/j.pdpdt.2020.102063 33068820

[5] Volgenant CMC, Fernandez YMM, Rosema NAM, van der Weijden FA, Ten Cate JM, van der Veen MH. Comparison of red autofluorescing plaque and disclosed plaque-a cross-sectional study. Clin Oral Investig. 2016;20(9):2551–8. doi: 10.1007/s00784-016-1761-z 26993658 PMC5119843

[6] Carter K, Landini G, Walmsley AD. Automated quantification of dental plaque accumulation using digital imaging. J Dent. 2004;32(8):623–8. doi: 10.1016/j.jdent.2004.06.006 15476956

[7] Michou S, Vannahme C, Ekstrand KR, Benetti AR. Detecting early erosive tooth wear using an intraoral scanner system. J Dent. 2020;100:103445. doi: 10.1016/j.jdent.2020.103445 32750388

[8] Marro F, Jacquet W, Martens L, Keeling A, Bartlett D, O’Toole S. Quantifying increased rates of erosive tooth wear progression in the early permanent dentition. J Dent. 2020;93:103282. doi: 10.1016/j.jdent.2020.103282 32006669

[9] Smith RN, Rawlinson A, Lath DL, Brook AH. A digital SLR or intra-oral camera: preference for acquisition within an image analysis system for measurement of disclosed dental plaque area within clinical trials. J Periodontal Res. 2006;41(1):55–61. doi: 10.1111/j.1600-0765.2005.00841.x 16409256

[10] Doi K, Yoshiga C, Kobatake R, Kawagoe M, Wakamatsu K, Tsuga K. Use of an intraoral scanner to evaluate oral health. J Oral Sci. 2021;63(3):292–4. doi: 10.2334/josnusd.21-0048 34108300

[11] You W, Hao A, Li S, Wang Y, Xia B. Deep learning-based dental plaque detection on primary teeth: a comparison with clinical assessments. BMC Oral Health. 2020;20(1):141. doi: 10.1186/s12903-020-01114-6 32404094 PMC7222297

[12] Rustogi KN, Curtis JP, Volpe AR, Kemp JH, McCool JJ, Korn LR. Refinement of the Modified Navy Plaque Index to increase plaque scoring efficiency in gumline and interproximal tooth areas. J Clin Dent. 1992;3(Suppl C):C9–12. 1306676

[13] Ramfjord SP. Indices for the prevalence and incidence of periodontal disease. J Periodontol. 1959;30(1):51–9.

[14] Landis JR, Koch GG. The measurement of observer agreement for categorical data. Biometrics. 1977;33(1):159–74. 843571

[15] Tran DT, Gay I, Du XL, Fu Y, Bebermeyer RD, Neumann AS, et al. Assessing periodontitis in populations: a systematic review of the validity of partial-mouth examination protocols. J Clin Periodontol. 2013;40(12):1064–71. doi: 10.1111/jcpe.12165 24192071 PMC3859863

[16] Goldberg P, Matsson L, Anderson H. Partial recording of gingivitis and dental plaque in children of different ages and in young adults. Community Dent Oral Epidemiol. 1985;13(1):44–6. doi: 10.1111/j.1600-0528.1985.tb00419.x 3855735

